# Rank Protein Immunolabeling during Bone-Implant Interface Healing Process

**DOI:** 10.1155/2010/513461

**Published:** 2010-07-19

**Authors:** Francisley Ávila Souza, Thallita Pereira Queiroz, Eloá Rodrigues Luvizuto, Renato Sussumu Nishioka, Idelmo Rangel Garcia-JR, Paulo Sérgio Perri de Carvalho, Roberta Okamoto

**Affiliations:** ^1^Department of Surgery and General Clinical, Araçatuba Dental School, Universidade Estadual Paulista, UNESP, Rua José Bonifácio 1190, Bloco 10A, Vila Mendonça, 16015-050 Araçatuba, Brazil; ^2^Department of Dental Materials and Prothesis, São José dos Campos Dental School, Universidade Estadual Paulista, UNESP, Brazil

## Abstract

The purpose of this paper was to evaluate the expression of RANK protein during bone-healing process around machined surface implants. Twenty male Wistar rats, 90 days old, after having had a 2 mm diameter and 6 mm long implant inserted in their right tibias, were evaluated at 7, 14, 21, and 42 days after healing. After obtaining the histological samples, slides were subjected to RANK immunostaining reaction. Results were quantitatively evaluated. *Results*. Immunolabeling analysis showed expressions of RANK in osteoclast and osteoblast lineage cells. The statistical analysis showed an increase in the expression of RANK in osteoblasts at 7 postoperative days and a gradual decrease during the chronology of the healing process demonstrated by mild cellular activity in the final stage (*P* < .05). *Conclusion*. RANK immunolabeling was observed especially in osteoclast and osteoblast cells in primary bone during the initial periods of bone-healing/implant interface.

## 1. Introduction

Of the significant advances in dentistry during the twentieth century, unquestionably none has extended the treatment horizons more than the successful use of osseointegrated implants. Applying the principles of osseointegration, dental surgeons are predictably able to replace missing teeth with excellent long-term esthetic and functional results [1].

Bränemark et al. [2] have defined osseointegration as a direct structural and functional connection between ordinary healthy bone and the implant surface, as seen at the level of optical microscopy, producing stability and allowing the structure to support the masticatory forces. A release of growth factors takes place right after an implant is inserted, which stimulates osteoblast precursors to develop into mature osteoblasts with consecutive production of bone tissue [3]. The process of bone remodeling, which involves many cellular steps and is not yet well understood, has been considered a repetition of the osteogenic lineage response that occurs in the fetus during the developmental phases [4].

The initial step in the bone-healing process starts with the migration of osteogenic cells, derived from the medullary bone layer, including undifferentiated mesenchymal cells, osteocytes, and osteoblasts, directly to a framework supported by blood a clot [5, 6]. The anabolic model is the first response of bone healing after implant placement in the cortical bone, similar to the process that occurs in fracture healing [7]. Thus, biologically, there is no evidence of complete contact between bone and the titanium surface, but the aspect considered is the greater or lesser amount of connective tissue, without clinical failure or fault of the implant [8]. In machined-surface implants, there is a larger quantity of conjunctive tissue in the initial stages of the repair process, when compared with porous-surface implants. 

Therefore, the repair process around machined-surface implants is time-dependant [9, 10]. This occurs because the implant surface has a smaller area of contact [11]. It is believed that retention of the blood clot is less stable, and consequently the migration of cells of osteoblastic origin occurs more slowly on machined surface implants. Therefore, the bone-interface contact in machined-surface implants is smaller in the initial stages of the repair process when compared with porous surface implants [9, 12]. 

The recent discovery of the proteins of the TNF family members has shown their roles in bone dynamics [13–16]. From these proteins, RANK is expressed in preosteoclast lineage cells [16], controlling bone resorption [17], and consecutively, calcium metabolism. The function of RANK protein is related to two other bone-matrix proteins, RANKL (receptor activator of nuclear factor kappa B ligand) and OPG (osteoprotegerin) [18]; the interaction among them will modulate bone turnover.

Considering the involvement of RANK, a TNF family member receptor-activator of nuclear factor kappa B, in bone turnover as the main pathway to achieve bone homeostasis, the purpose of this paper was to evaluate RANK immunolabeling during the bone-implant interface healing process during different periods of its chronology.

## 2. Material and Methods

### 2.1. Animals

Twenty male Wistar rats, weighing between 300 and 350 g, 90 days old were maintained at a temperature of 22 8C, in a 12-h light/12-h dark cycle, *ad libitum* to water and rat food. The principles of laboratory animal care (NIH publication 85-23, 1985) [19] and national laws on animal use were complied with in the present study, which was authorized by the Animal Research Ethics Committee of the São Paulo State University, Brazil (Protocol Number 36/05). 

### 2.2. Surgical Procedure

The animals received general anesthesia with xylazine (0.03 mL/100 g bw/im—Dopaser Laboratories Calier S.A., Barcelona, Spain) and Ketamine (0.07 mL/100 g bw/im—Fort Dodge Saúde Animal Ltda, Brazil). After trichotomy and antisepsis (Polyvinylpyrrolidone iodide; Indústria Química e Farmacêutica Rioquímica Ltda, Brazil) of the right tibia, a dermoperiosteal incision was performed in the lateral view of right tibia in order to gain surgical access. The osteotomy was performed with a specific drill, 2 mm in diameter (with an internal hexagon 1.2 mm in diameter) (SIN—Sistema Nacional de Implants, São Paulo Brazil) and implant placement was performed with a digital key 1.17 (SIN, Sistema de Implante Nacional, São Paulo, Brazil) which fitted into the 1.2 mm diameter of the internal hexagon of the implant).

The tissue was sutured in different plans, using polylactic acid thread (Vycril 4.0, Ethicon, Johnson Prod., São José dos Campos, Brazil) in the deep layer and nylon in the superficial plane (Nylon 5.0, Mononylon, Ethicon, Johnson Prod., São José dos Campos, Brazil). In addition, animals received a single dose of 20,000 UI of penicillin G benzathine (Fontoura Wyeth S.A.) by intraperitoneal injection and were divided into four groups to allow analysis of the wound healing process at 7, 14, 21, and 42 days.

### 2.3. Collection of Materials

After the experimental periods, the animals were anaesthetized and infusion with 4% formaldehyde (Acros Organics, New Jersey, USA), was performed using a Masterflex LS perfusion pump (Cole-Parmer Instrument Company, Vermont Hills, IL, USA), to remove the right tibia. The bone blocks were postfixed in 4% formaldehyde, demineralized in 5% EDTA (Merck, Darmstadt, Germany) and cryoprotected in sucrose (Merck, Darmstadt, Germany). After this, the implants were removed with the use of a 1.17 key (SIN, Sistema de Implante Nacional, São Paulo, Brasil) which was carefully fitted into the internal hexagon of the implant, so as not to cause injury to the bone tissue around the implants. Therefore, they were removed after the decalcification process, and it may also be mentioned that in the act of inserting the implant, it was developed especially for this study, with the dimensions already described and with an internal hexagon 1.20 mm in diameter.

 Transversal sections to the area corresponding to the implants were cut on a cryostat (Micron Zeiss, Berlin, Germany) to obtain 14 mm thick slices, thin enough to allow an immunohistochemical analyis, which were mounted on previously gelatinized slides. 

### 2.4. Immunohistochemical Processing

An anti-RANK primary antibody was used (Rabbit anti-RANK polyclonal-Santa Cruz Biotechnology, California, USA). As a secondary antibody, a biotinylated donkey antirabbit antibody (Jackson Immunoresearch Laboratories, West Grove, Pennsylvania, USA) was used. The immunohistochemical reaction was amplified with an avidin biotin system (Kit ABC- Vectastain Elite ABC—Peroxidase Standard, reagent A and B only—PK6100—Vector Laboratories, Burlingame, CA, USA) and diaminobenzidine (Sigma Aldrich, St Louis, Missouri, USA) was used as chromogen. Immunohistochemical reactions were controlled to evaluate the specificity of the labels omitting the primary antibody (negative controls). The analyses were performed without the knowledge of the examiner that was well calibrated. Positive control was performed in the nasal cavity of rats for the osteoclast and in primary bone of newborn rats for the osteoblast. Negative control was performed by omitting the primary antibody to see the veracity of the reaction. 

 Hematoxylin and eosin staining was performed and used as a reference of the cytoarchiteture of the tissue sides of the immunohistochemistry reactions; some slides were stained with Hematoxylin and eosin in order to receive the cytoarchitecture orientation. Data analysis was performed in a semiquantitative manner, with scores ranging from “−” for absence of marking and “+, ++ and +++” for little, medium, and a great deal of marking, respectively.

The transversal sections allowed visualization of the bone tissue formed in contact with the implant. The titanium implants were removed from the samples after the demineralization process was complete. Therefore, the area analyzed was that around the negative area of the implant. To facilitate comparisons, scores were converted into percentile averages frequencies of 0%, 20% (10% to 30%), 60% (50% to 70%), and 90% (*0% to 100%). The results obtained considering the expression of RANK in osteoblasts (GI) and RANK in osteoclasts (GII) were joined, tabulated for analysis, and submitted to the Mann-Whitney test to compare each group with each period, and to the Kruskal-Wallis and Dunn Multiple Comparison nonparametric tests (*α* = 5%) to compare each group in all periods. The cells stained by immunohistochemistry, which were shown to be multinucleated were considered to be osteoclasts, and those around or within the bone trabeculae that were not multinucleated, were considered osteoblasts.

## 3. Results

### 3.1. Clinical Analysis

None of the implants failed. All the implants were stable, without loss of the surrounding bone.

**Qualitative Imunohistochemistry Analysis**

For imunohistochemistry analysis, the imunolabelings taken into consideration were those observed in the osteoclasts, osteoblasts, and osteocytes in neoformed bone, around the implant (negative area).

**Figure 2:**
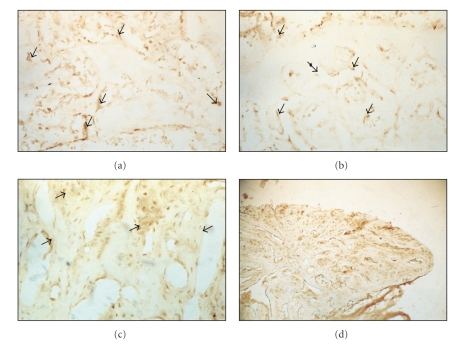
RANK immunolabeling during bone-implant interface healing process during 7 (a), 14 (b), 21 (c), and 42 (d) days postoperative.

At 7 postoperative days, neoformed trabecular bone tissue was observed with osteoblasts located around it synthesizing bone matrix. In addition, connective tissue was observed in some areas close to the implant. RANK protein expression was observed in osteoblast lineage cells (Figure 2(a)). 

At 14 postoperative days, a larger quantity of bone formation was observed. RANK immunolabeling was observed in osteocyte phenotypes and bone-lining cells. There was no expression in osteoblast phenotype cells in this period (Figure 2(b)). At 21 postoperative days, RANK expression was reduced when compared with previous periods and the morphology of the cells differed from the aspect observed at 7 postoperative days (Figure 2(c)). Dispersed trabecular bone was also observed, some cells with an aspect of bone-lining cells or latent osteocytes, with no metabolic activity. In some areas, it was possible to observe RANK immunolabeling in macrophages or preosteoclasts. At 42 postoperative days, matured bone trabeculae were observed (Figure). RANK expression was not present (Figure 2(d)).

### 3.2. Controls

The negative and positive controls, showed the specificity of the reactions applied. The positive controls were performed in slices from the rat nasal cavities, an area in which RANK expression is observed in macrophages, showing the specificity of the antibody used in the immunolabeling of preosteoclast lineage cells.

**Figure 3:**
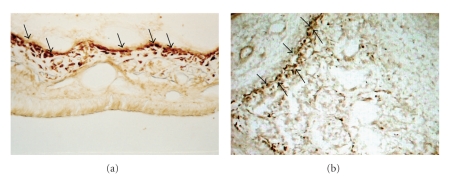
Positive controls from RANK expression. (a) Rats nasal cavity showing RANK expression in preosteoclasts lineage cells. (b) Rat embryo with 21 days of pregnancy. A bodily structure that expresses just osteoblasts lineage cells in primary bone tissue is the palatine process.

In addition, to evaluate the possibility of RANK protein expression in osteoblast-lineage cells, immunohistochemical reactions were performed in the rat embryo at 21 days of pregnancy. A bodily structure that expresses only osteoblast-lineage cells in primary bone tissue is the palatine process. This process was also analyzed as a positive control and showed positive immunolabeling for RANK protein (Figure 3). The immunomarkings shown in Image 3 refer to the RANK immunomarkings in areas outside of the studied area, functioning as positive control to show the effectiveness of the antibody used. Figure 3(a) shows the RANK expression in osteoclasts of the nasal mucosa of rats, and Figure 3(b) shows the RANK expression in osteoblasts in the primary bone tissue of rat embryos. These two regions outside of the studied area are regions that are known to have a large quantity of osteoclasts in the nasal cavity and a large quantity of osteoblasts in rat embryos. Therefore, the specificity of the antibody used is confirmed.

**Quantitative Imunohistochemistry Analysis**

**Figure 4:**
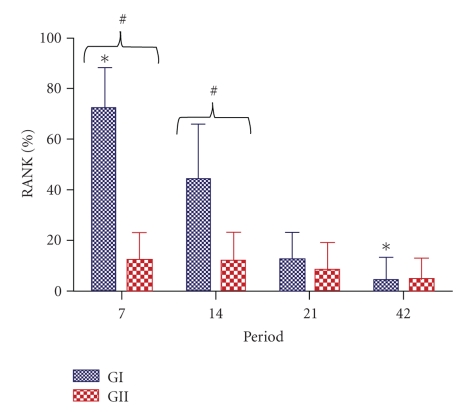
Comparison among RANK percentile values for each group. GI: RANK immunolabeling in osteoblasts lineage cells; GII: RANK immunolabeling in osteoclasts lineage cells.

Figure 4 shows the mean values (M) and the standard deviation (SD) for GI and GII during different periods analysed.

Statistically significant difference was observed between GI and GII at 7 and 14 postoperative days (*P* =  .0109; and  .0420, resp.). Significant difference was also observed between GI at 7 and GI at 14 days (*P* =  .0012) (Figure 4).

## 4. Discussion

Several aspects must be considered in the results observed. Immunohistochemistry is a methodological approach that was recently introduced in the study of the bone-repair process [20]. Since then, it has been used consistently, as it allows the proteins present in cells to be identified, thus contributing to a better understanding of bone biology.

The use of frozen sections was chosen, as this type of procedure allows a greater preservation of tissue antigenicity, giving better results of the labels observed. Therefore, it must be considered that positive and negative controls were performed with the aim of evaluating the absence of unspecific labels, as well as specificity of labels in the primary antibody used.

The interface formed between bone and implant has been most studied and discussed by many research centers. Considering that human bone tissue undergoes complete reconstitution of the previous stage of injury, Walter and Talbot [21] used the expression regeneration, instead of repair process. Moreover, the regeneration process at the interface occurs in the following three steps: hemostasis, granulation tissue formation, and bone formation [22, 23].

The primary function of the interface formed between the bone and implant is to provide a safe and efficient charge transfer through the implant to the bone tissue [24]. Many studies have determined the factors that interfere in this interface, among them, the type of surface, showing that the different surface treatments have positive effects on the initial bone repair in animals [25–28] and in humans [29]. Therefore, the purpose of this study was to evaluate the machined surface implants, since the primary aim was to evaluate the cellular responses during the osseointegration process, considering RANK expression and not to compare the topographical modifications of implant surfaces.

The results showed that RANK protein was expressed in different ways during the analyzed periods. The manner that it was expressed was related to the dynamics of the bone remodeling process. Studies have shown that RANK is a receptor protein expressed in macrophages of the osteoclast lineage, dendritic cells, and fibroblasts [14, 30, 31]. In the analyzed results, it was observed that, particularly in the 21 postoperative days, there were some cells with an aspect similar to that of osteoclastic macrophages presented RANK protein immunolabeling, as described in literature [14, 30, 31]. Furthermore, at 7 days after implant placement, RANK protein immunolabeling was observed in cells with a similar morphology to that of osteoblasts and was located around neoformed bone tissue. It was also observed in some areas, the immunolabeling of cells similar to osteocytes, locked into bone tissue lacunae was also observed. The RANK immunolabeling cells with the aspect of osteoblasts, which presented intense activity, were observed in all animals at 7 postoperative days. 

A hypothesis that could explain the expression of RANK protein in cells similar to osteoblasts would be that this receptor protein is present in this type of cell, but it is located in a less exposed manner that makes it difficult to label. Thus, when there is a stimulus such as in the osseointegration process that evokes tissue preparation to form bone around the inserted implant, this receptor could change its conformation, becoming more exposed in the cell membrane, and therefore positive to immunolabeling.

The presence of RANK in cells with osteoblast fenotypes could be justified based on the hypothesis that osteoblasts are cells that present autocrine regulation, or that have receptors of their secretion products. Since the osteoblasts synthesize, it is possible that they must have the receptor of this ligand or RANK protein. Pettit et al. [32] during the evaluation of articular bone erosion in rheumatoid arthritis observed RANK expression in cells that had morphological features of osteoblasts. Furthermore, It must be considered that in our positive control experiments, the RANK immunolabeling was observed in primary bone of the palatine process of an embryo at the end of pregnancy, showing that the RANK protein or the RANK ligand receptor is present in osteoblasts.

With regard to the temporal analysis of the osseointegration process, it was observed that after 14 postoperative days, there was a decrease in the expression of RANK proteins in osteoblasts lineage cells, and at 14 and 21 postoperative days, it was observed the RANK immunolabeling in osteocytes and bone-lining cell. At 42 days postoperative, there was no expression of RANK protein in the bone tissue.

The immunohistochemistry approach could raise doubts with regard to the observed results. Therefore, the control reactions confirmed the observed labelings. 

Similar results were observed in our laboratory (paper submitted for publication) when RANK expression in osteoblasts during the alveolar healing process in rats. Coincidentally, the larger RANK protein expression in osteoblasts was at 14 days postoperative, a period market by greater metabolic activity of the cells during the bone healing process.

The results presented herein showed that osteoblast-lineage cells express RANK protein in primary bone during the initial periods of the bone-healing process around titanium implants. 

## Figures and Tables

**Figure 1:**
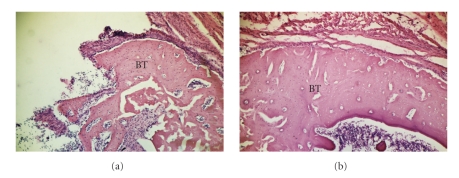
Hematoxylin and eosin staining as a reference of the cytoarchiteture of the tissue. ((a) 63x; (b) 160x). Bone tissue formed around the implant at 21 days postoperative.
